# 

**Published:** 1896-02-22

**Authors:** 


					*r7HmUSFTTZ.L. ~ ?-
ABSOLUTELY PURE, tberftfor* BEST February 22nd, 1896.
CADBURYS ^ CADBURY'S
COCOA
1 The standard of highest purity at present
attainable."?Lanett.
S0COA
NO ALKALIES USED. ~ . -
S. Q
LASConr Westerh Infirmar*
1
Sf
'RWICH HOSP.LTAl ]
No. 491. Vol. XIX.
Saturday,
Feb. 22nd, 1896.
WITH EXTRA NURSING SUPPLEMENT. ^1KSL.S??5B2"-
[Registered at the General Post Offioe as a Newspaper for Monthly Parts, 6d.j post free, 8&,
transmission in the United Kingdom and Abroad.] Ditto per Annum, 8s.6d., post free.

				

